# Psychological distress is affected by fear of COVID-19 via lifestyle disruption and leisure restriction among older adults in Japan: a cross-sectional study

**DOI:** 10.3389/fpubh.2023.1264088

**Published:** 2023-10-30

**Authors:** Yosuke Zenba, Akihiro Kobayashi, Tadanori Imai

**Affiliations:** ^1^Department of Occupational Therapy, School of Allied Health Sciences, Kitasato University, Kanagawa, Japan; ^2^Department of Occupational Therapy, Faculty of Rehabilitation, Gunma University of Health and Welfare, Gunma, Japan

**Keywords:** leisure activities, psychological distress, lifestyle, COVID-19, healthy aging

## Abstract

**Introduction:**

Engaging in social activities is an essential component of a healthy lifestyle for community-dwelling older adults. Critically, as with past disasters, there is concern about the effects of long-term activity restrictions due to the coronavirus disease 2019 (COVID-19) pandemic on health of older adults. However, the precise associations between fear of COVID-19, lifestyle satisfaction, leisure activities, and psychological distress are unclear.

**Objective:**

The purpose of this study was to comprehensively determine the associations between fear of COVID-19, lifestyle satisfaction, leisure engagement, and psychological distress among community-dwelling older adults in the context of the ongoing COVID-19 pandemic.

**Materials and methods:**

A questionnaire survey administered by mail was conducted from October 1 to October 15, 2021. The questionnaire included the Fear of COVID-19 Scale, the Lifestyle Satisfaction Scale, the Leisure Activity Scale for Contemporary Older Adults, and the Kessler Psychological Distress Scale-6. Based on previous studies, we developed a hypothetical model for the association between fear of COVID-19, lifestyle satisfaction, leisure engagement, and psychological distress and performed structural equation modeling to assess the relationships between these variables.

**Results:**

Participants included 301 Japanese citizens (23.6% male, 76.4% female), with a mean age of 76.7 ± 4.58 years. Goodness-of-fit from structural equation modeling was generally good. Analysis of standardized coefficients revealed a significant positive relationship between fear of COVID-19 and psychological distress (β = 0.33, *p* < 0.001) and lifestyle satisfaction and leisure activities (β = 0.35, *p* < 0.001). We further observed a significant negative relationship between fear of COVID-19 and lifestyle satisfaction (β = −0.23, *p* < 0.001) and between leisure activities and psychological distress (β = −0.33, *p* < 0.001).

**Conclusion:**

Fear of COVID-19 is significantly associated with psychological distress, both directly and via its effects on lifestyle satisfaction and leisure activities. That is, not only did fear of COVID-19 directly impact psychological distress of participants, it also affected psychological distress through lifestyle disruption and leisure restriction. This results may be used to better understand how a national emergency that substantially restricts daily life, such as COVID-19 or an earthquake disaster, can affect the psychological health and wellbeing of older, community-dwelling adults.

## 1. Introduction

Japan is one of the most rapidly aging societies in the world, with a life expectancy at birth of 81.5 years for Japanese men and 86.9 years for women in 2019. In contrast, healthy life expectancy at birth is only 72.6 years for men and 75.5 years for women (1), approximately 9 years shorter than average life expectancy for men and 11 years shorter for women. Given that healthy life expectancy refers to the period during which a person can live a healthy life without any hindrance in daily living, there is growing emphasis on extending this period to improve quality of life for older individuals.

Studies have shown that for older adults dwelling in communities, having a sense of Ikigai (i.e., purpose that gives life meaning) through leisure activities is important for leading a healthy life (2, 3). In particular, the health hazards associated with restricted social activities have been clearly demonstrated after past disasters, such as the Great East Japan Earthquake in 2011 (4–6). Accordingly, there is substantial concern about the effects of long-term activity restrictions on health of older adults during the ongoing coronavirus disease 2019 (COVID-19) pandemic.

COVID-19 was first reported in Wuhan, China, in December 2019 and has since spread across the globe, significantly impacting society at every level (7–9). In Japan, the first case of severe acute respiratory syndrome coronavirus 2 (SARS-CoV-2) infection was confirmed on January 15, 2020, after which the disease was rapidly disseminated throughout the country. To slow the spread in infection, the first state of emergency was declared for Tokyo, Saitama, Chiba, Kanagawa, Osaka, Hyogo, and Fukuoka on April 7, 2020. The scope of application subsequently expanded nationwide, and the decree was lifted on May 25 of the same year. However, in response to the ongoing evolution of the pandemic and spread of COVID-19, a total of three states of emergency (April–May 2020, January–March 2021, and April–September 2021) were declared in the following years, depending on the region. Unlike lockdowns in other countries, the state of emergency declaration in Japan is not mandatory but rather is only a request for self-restraint. Even so, the emergency declarations and the COVID-19 pandemic, in general, have had a significant impact on people's lives, forcing them to change their conventional lifestyles, such as by refraining from going out and working from home, and resulting in the closure of educational institutions, restaurants, and stores (10).

To prevent the continued spread of COVID-19, it is important to reduce human contact as much as possible (11, 12). To this end, public health experts have recommended the “new normal,” which includes staying home, keeping physical distance, and avoiding the 3Cs: closed spaces, crowded places, and close-contact settings. Because older adults, in particular, are more susceptible to severe COVID-19 (13), it is critical for these individuals to avoid infection. However, the resulting reduction in interpersonal contact has also decreased opportunities for social and leisure activities that older individuals used to engage in. These changes in daily life, such as limiting or refraining from social activities, can have a significant impact on the physical and mental health of older adults.

Since the early stages of the pandemic, efforts have been aimed at assessing the effects of COVID-19 on mental and physical functions, with several published reports focusing on social activities and mental health of older adults. Studies measuring social activities have noted a decrease in physical activity (14), interpersonal interaction (15), and engagement in leisure activities (16) due to a long period of self-restraint. In parallel, mental health studies have reported increased fear and anxiety about contracting SARS-CoV-2, which is at the core of the problem, as well as secondary effects of depression and anxiety due to restricted activity (17–19). Collectively, these findings reveal that the mental health of older adults in response to COVID-19 is affected by a variety of factors. Therefore, to fully understand the effects of the pandemic on mental health, it is necessary to comprehensively consider not only the fear of COVID-19 but also lifestyle changes, engagement in leisure activities, and other factors. However, to our knowledge, previous studies have only examined each factor separately, focusing primarily on one-to-one interactions, and none has comprehensively examined the causal relationships between fear of COVID-19, lifestyle satisfaction, leisure activities, and psychological distress.

The aim of this study was to determine the association between fear of COVID-19, lifestyle satisfaction, leisure activities, and psychological distress among community-dwelling older adults in the context of the ongoing COVID-19 pandemic. For this purpose, we developed hypothetical models for each of these variables, in which we hypothesized that the fear of COVID-19 would influence psychological distress. In addition, we expected that fear of COVID-19 would affect the participants' lifestyle satisfaction and leisure activities, such as social roles and routines, and that the inability to engage in prior activities would exacerbate psychological distress. Overall, we anticipate that this study may provide useful information for understanding the impacts on older individuals when social activities are restricted by new infectious disease outbreaks or future major disasters, such as earthquakes.

## 2. Materials and methods

### 2.1. Participants and procedure

This study is part of an ongoing cohort study since 2007. A questionnaire survey administered by mail was conducted from October 1 to October 15, 2021. Subsequently, 498 older adults participated in this survey after attending a workshop project sponsored by the Ibaraki Prefecture in Japan. Participation in the study was voluntary and anonymous, and participants could withdraw from the study at any time. Approval for this research was obtained from the Research Review and Ethics Committee of the affiliated institution, and protection of personal information and research consent procedures were carried out in accordance with ethical regulations. The purpose and content of the study was explained to the participants in a written form, and they were considered to consent by filling out the survey.

The questionnaire included the Japanese version of the Fear of COVID-19 Scale (FCV-19S), the Lifestyle Satisfaction Scale, the Leisure Activity Scale for Contemporary Older Adults (LASCO), and the Japanese version of the Kessler Psychological Distress Scale-6 (K6). Scale details are outlined in the following section. Demographic information collected included participants' age, gender, marital status, and living situation, as well as self-rated health status (1, very good; 2, good; 3, somewhat poor; 4, poor), outpatient treatment, financial situation, and frequency of going out (less or more than once per week). The 301 participants who responded in person and had no missing variables in their responses were included in this analysis.

### 2.2. Measures

#### 2.2.1. Fear of COVID-19 scale

The Japanese version of the FCV-19S was used to assess fear of COVID-19 (20). The FCV-19S is a self-administered questionnaire that measures fear of novel coronaviruses and requires responses to seven questions using a five-point scale ranging from 1 (not at all applicable) to 5 (very applicable). Higher scores indicate greater fear of COVID-19. The FCV-19S is used worldwide because it is easy to measure, and its reliability and validity have been verified in Japan (21–23).

#### 2.2.2. Lifestyle Satisfaction Scale

This item aimed to investigate lifestyle disruption caused by the COVID-19 pandemic, but an appropriate rating scale could not be found. Therefore, we asked participants to respond to their current lifestyle (i.e., habits, routines, and roles) using a four-point scale, ranging from 1 (not satisfied) to 4 (very satisfied), as described in previous studies (24). Higher scores indicate greater lifestyle satisfaction, and lower scores indicate lifestyle disruption.

#### 2.2.3. The leisure activity scale for contemporary older adults

This instrument was used to assess engagement in leisure activities (25). The survey consists of 11 items (i.e., Technology Use, Social–Public, Social–Private, Physical, Developmental, Cultural, Travel, Creative, Raising Plants, Intellectual Games, and Competitive Games), and participants were asked to indicate their implementation status using a four-point scale, ranging from 0 (not at all) to 3 (often). The reliability of this scale has been confirmed for older adults living in urban areas of Japan (25).

#### 2.2.4. The Kessler psychological distress scale-6

We measured psychological distress using the Japanese version of the K6 (26, 27). Participants were asked to respond to six questions using a five-point scale, ranging from 0 (not at all) to 4 (always). The total score ranges from 0 to 24, with higher scores indicating greater distress. This instrument has been used in many studies to screen for mental disorders and psychological distress in population health studies.

### 2.3. Statistical analysis methods

All analyses were performed using SPSS28.0 and Amos29.0 software. For each scale, a factor analysis was conducted to examine its structure and structural validity. In this factor analysis, items with factor loadings <0.4 were deleted, and Cronbach's alpha scores were also calculated to check for internal consistency. Correlation coefficients were then calculated to compute descriptive statistics and examine associations between variables. In addition, structural equation modeling was performed based on the hypothetical model created in this study. The following goodness-of-fit indices were used to evaluate the degree of fit: Tucker–Lewis index (TLI) > 0.90, comparative fit index (CFI) > 0.90, and root mean square error of approximation (RMSEA) <0.06 (28). The level of significance was set at *p* < 0.05.

## 3. Results

### 3.1. Characteristics of study participants

Demographic information for the study participants is shown in Table 1. Participants include 301 older Japanese adults (23.6% male, 76.4% female) living in the Ibaraki prefecture, with a mean age of 76.7 ± 4.58 years; 76% of participants were married at the time of survey completion, and 82.3% lived with a cohabitant. Although 82.9% of participants had regular outpatient treatment, 87.7% reported their health status as relatively healthy. In addition, 83.6% of participants were financially comfortable, and 95% went out at least once a week. Of all participants, 47.0% lived in urban areas and 39.9% in rural areas.

**Table 1:** Demographic characteristics of study participants (*n* = 301).

**Variables**	**Frequency (*n*)**	**Percentage (%)**
**Gender**		
Male	71	23.6
Female	230	76.4
**Age (years)**		
<66	3	1.0
66–70	27	9.0
71–75	81	26.9
76–80	132	43.8
>80	58	19.3
**Education**		
≤ 12 years	192	64.4
≥13 years	106	35.6
**Married status**		
Unmarried/Divorced/Widowed	72	24.0
Married	228	76.0
**Residential area** ^*^		
Urban	140	47.0
Rural	119	39.9
Others	39	13.1
**Living situation**		
Alone	52	17.7
With spouse	163	55.4
With children	79	26.8
**Outpatient treatment**		
No	51	17.1
Yes	248	82.9
**Subjective financial situation**		
Uncomfortable	49	16.4
Comfortable	250	83.6
**Self-rated health status**		
Very good	41	13.7
Good	222	74.0
Somewhat poor	28	9.3
Poor	9	3.0
**Frequency of going out**		
Less than once a week	15	5.0
More than once a week	284	95.0

^*^As of responses from the 2007 survey.

### 3.2. Examination of scales structures and correlation coefficients

We first conducted an exploratory factor analysis on each scale to examine the factor structure of the scales. For the FCV-19S, one item suspected of multicollinearity was deleted, and six items with one factor were used. Seven items from the LASCO with a factor loading of 0.4 or higher were retained. For the K6, six items with one factor were retained without deleting any item. All Cronbach's alpha scores are shown in Table 2. We then performed correlation analysis with the FCV-19S, Lifestyle Satisfaction Scale, LASCO, and K6; results are presented in Table 2. Correlations were found between all measures except the FCV-19 and LASCO.

**Table 2:** Means, standard deviations, and Cronbach's alpha scores for the FCV-19S, lifestyle satisfaction, LASCO, and K6 and correlations between measures.

	**Mean (SD)**	α^*^	**FCV-19S**	**Lifestyle**	**LASCO**
FCV-19S	16.38 (4.28)	0.827			
Lifestyle	2.97 (0.57)		−0.184^**^		
LASCO	10.69 (3.92)	0.776	−0.046	0.248^**^	
K6	2.95 (3.18)	0.845	0.332^**^	−0.288^**^	−0.210^**^

^*^Cronbach's alpha. ^**^p < 0.01. FCV-19S, Fear of COVID-19 Scale; Lifestyle, Lifestyle Satisfaction Scale; LASCO, Leisure Activity Scale for Contemporary Older Adults; K6, Kessler Psychological Distress Scale-6. Correlation: Spearman's rank correlation.

### 3.3. Structural equation modeling

**Figure 1:**
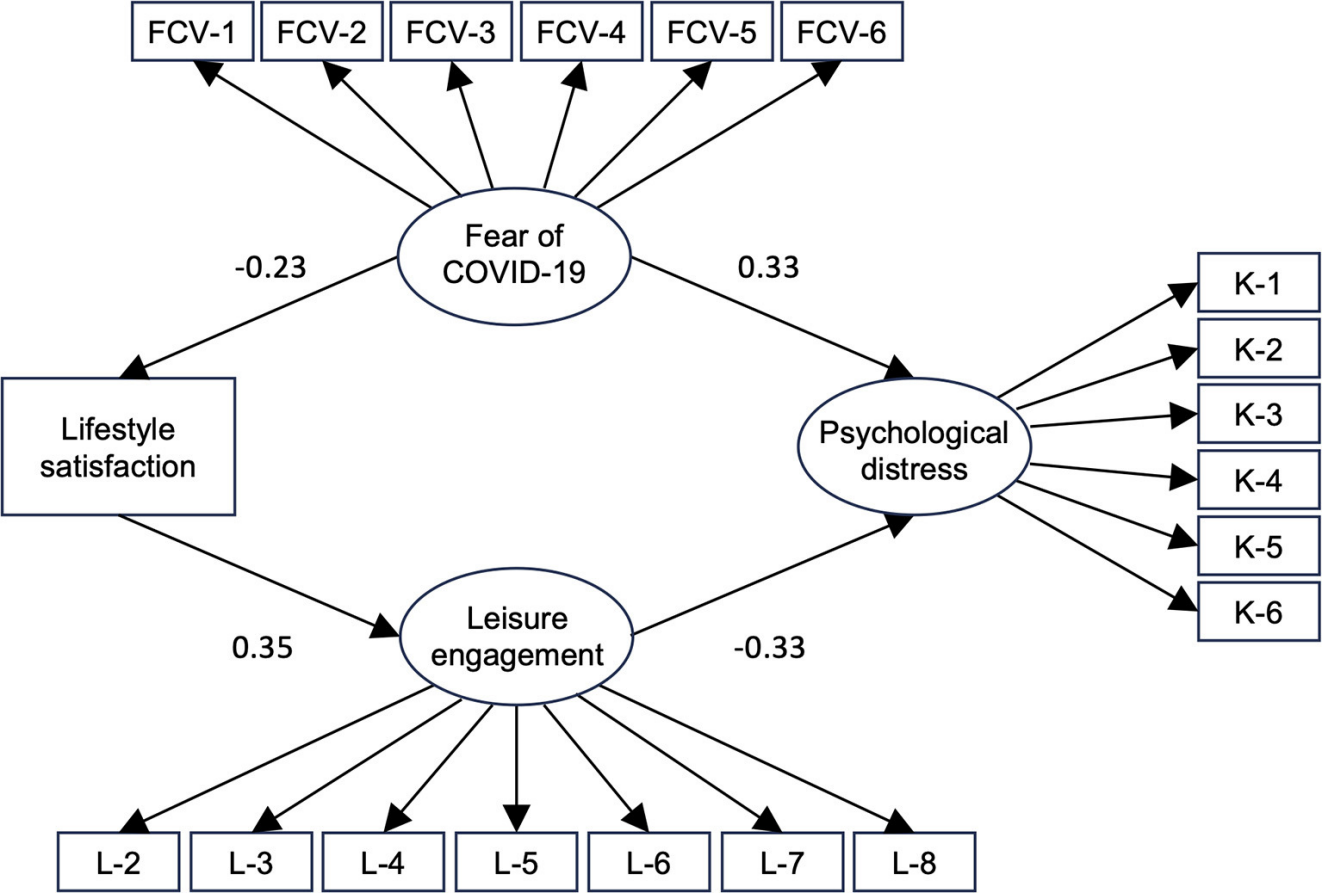
Structural equation modeling results. Standardized coefficients are shown in the figure. Lifestyle satisfaction was measured using the Lifestyle Satisfaction Scale created for this study.

Structural equation modeling was performed to examine the relationships among the variables (Figure 1). Goodness-of-fit indices were as follows: TLI = 0.936, CFI = 0.946, and RMSEA = 0.047, indicating an acceptable level of fit. Standardized coefficients showed a significant positive relationship between fear of COVID-19 and psychological distress (β = 0.33, *p* < 0.001) and lifestyle satisfaction and leisure activities (β = 0.35, *p* < 0.001). We further detected a significant negative relationship between fear of COVID-19 and lifestyle satisfaction (β = −0.23, *p* < 0.001) and between leisure activities and psychological distress (β = −0.33, *p* < 0.001).

## 4. Discussion

### 4.1. Framework for supporting community life of the older adults

In Japan, “the community-based integrated care system” has been established to enable older adults to live their own way in their own neighborhoods. This model promotes health and care prevention through social participation such as leisure activities (29). Indeed, prior research has reported that taking part in social activities decreases risk of functional impairment, maintains cognitive function, and decreases prevalence of depressive symptoms (30–32). Therefore, it is important for community-dwelling older adults to be encouraged to engage in social activities.

### 4.2. Characteristics of study participants

Participants of this study included older adults in a resident-participating long-term care-prevention project. The mean age was 76.7 years, and the majority of participants were older than 75. The male/female ratio was 76.4%, with more females. In general, women are more likely to participate in care-prevention activities in the community, and this group was classified as active, with 95% of participants going out at least once a week. Study participants were gathered from various regions throughout the prefecture and are considered representative of community-dwelling older adults.

### 4.3. Fear of COVID-19 affects psychological distress

We found that fear of COVID-19 had a positive effect on psychological distress; that is, a stronger fear of COVID-19 led to increased psychological distress. Since March 2020, the lives of older adults have changed drastically relative to before the COVID-19 pandemic (33), resulting in various psychological problems. For example, fear and anxiety about contracting COVID-19 can have a significant impact on the mental health of older adults (34–36). Previous studies have similarly reported that fear of COVID-19 affects mental health. We note that our study was conducted in October 2021, approximately 1.5 years after the outbreak of SARS-CoV-2. Therefore, it is clear that fear of COVID-19 has a long-term impact on psychological distress. Another published study noted short-term effects on psychological wellbeing in the early stages of the COVID-19 pandemic, as well as long-term effects due to a prolonged period of self-restraint (37). Thus, our findings are similar to those reported in previous studies and support our hypothesis that fear of COVID-19 affects psychological distress.

### 4.4. Path from fear of COVID-19 to psychological distress via lifestyle disruption and leisure restriction

Because of their vulnerability and susceptibility to severe illness due to SARS-CoV-2 infection, older individuals have been asked to refrain from or limit their activities. Although these restrictions serve as important infection-prevention measures, prolonged self-restraints result in lifestyle changes and reduce opportunities for leisure and social activities. This new normal to prevent viral infection has had profound social, psychological, and physical effects. In particular, the negative impact of restricted leisure activities on mental health has been noted in several published studies (16, 38). Given that leisure activities are important for maintaining lifestyle satisfaction and good physical and mental health in older adults (39), there are also concerns about prolonged self-restraint leading to increased frailty, a condition referred to as Corona-Frailty (40). These observations highlight the need to comprehensively relationships among these factors in older adults.

This study is novel in that it revealed a comprehensive association between variables, rather than the one-to-one associations previously examined. Notably, we found that fear of COVID-19 affected psychological distress through effects on lifestyle satisfaction and engagement in leisure activities. Thus, these results suggest that fear of COVID-19 increases psychological distress by disrupting lifestyle and restricting engagement in leisure activities. Previous studies have noted a one-to-one association between fear of COVID-19 and psychological distress (35) and between engagement in leisure activities and mental health (16). However, a comprehensive association between fear of COVID-19 and psychological distress via lifestyle satisfaction and engagement in leisure activities is a new finding.

Restrictions associated with the new normal are required to reduce transmission during an infectious pandemic, such as COVID-19. However, given the ongoing nature of the pandemic, social activities for older adults have been restricted for a long period of time, and it has been difficult for these individuals to establish a new normal. Previous studies have reported many limitations for older adults in the wake of COVID-19, including those affecting leisure and social activities and interpersonal interactions (41–43). Results from this study suggest that failure to establish a new lifestyle that includes social and leisure activities will adversely affect the mental health of older individuals. Even before the COVID-19 pandemic, it was known that social and leisure activities are important for healthy living among older adults (30). Our results are consistent with these findings and suggest that safe social activities are important to prevent deterioration of mental health during the COVID-19 pandemic.

### 4.5. Clinical implications

The results of this study suggest that improving lifestyle satisfaction and engagement in leisure activities may prevent psychological distress among older adults. Thus, for those who have a strong fear of COVID-19, it is likely that psychological distress could be improved by providing support increase lifestyle satisfaction and enable engagement in safe leisure activities. Since 2020, the spread of SARS-CoV-2 has necessitated self-restraint and limitations in social interactions to prevent infection, and as a result, the lives of older adults have changed drastically. In response, there is increasing concern about the decline in physical and mental functions of older adults, with various reports describing Corona-Frailty (44–46) and a separate report claiming that fear of COVID-19 increases the risk of frailty in older adults (47).

Regardless of social circumstances, engaging in leisure and other activities has always been important for the health of older adults. Moreover, it is important for one's health to have a good living environment and to engage in leisure activities of one's choice. Thus, our findings indicate that following national emergencies, such as the COVID-19 pandemic, efforts to promote lifestyle restructuring and engagement in leisure activities are needed to maintain mental and physical health among community-dwelling older adults.

### 4.6. Limitations

We understand that this study has several limitations. First, our data are from a cross-sectional analysis conducted at a single point in time. Thus, without longitudinal data, we are unable to examine causal relationships over time. Second, the subjects were participants in a resident-participating long-term care-prevention project and are considered to be a group with high health consciousness and good physical and mental functions compared to their peers. Third, we assumed they are all Japanese based on their name, but ethnicity data was not included in the survey which may limit our implications. Lastly, the majority of participants were female. As gender differences in social and leisure activities have been reported, additional research is needed to investigate the effects of gender on the observed relationships.

## 5. Conclusions

We conducted a mailed questionnaire survey of community-dwelling older adults to investigate the relationship between fear of COVID-19, lifestyle satisfaction, leisure engagement, and psychological distress during the COVID-19 pandemic. We found that not only did fear of COVID-19 directly affect psychological distress, it also indirectly impacted psychological distress through lifestyle disruption and leisure restriction. These findings suggest it is necessary to focus on lifestyle and leisure engagement in order for older adults living in community settings to continue experiencing good mental and physical health, particularly following a national emergency, such as COVID-19 or an earthquake disaster, which can greatly restrict daily life. However lifestyle and leisure varies among different gender and personal preferences, and further study is necessary to provide tailored support for older adults.

## Data availability statement

The raw data supporting the conclusions of this article will be made available by the authors, without undue reservation.

## Ethics statement

The studies involving humans were approved by the Research Ethics Review Committee of Kitasato University School of Allied Health Sciences. The studies were conducted in accordance with the local legislation and institutional requirements. The participants provided their written informed consent to participate in this study.

## Author contributions

YZ: Conceptualization, Data curation, Formal analysis, Investigation, Methodology, Writing—original draft. AK: Investigation, Validation, Writing—review & editing. TI: Funding acquisition, Investigation, Supervision, Validation, Writing—review & editing.
